# Calculation of solar ultraviolet influx in the eye considering the field of view and pupillary dilation due to sunglasses

**DOI:** 10.1038/s41598-023-50831-9

**Published:** 2024-03-19

**Authors:** Mauro Masili, Fernanda O. Duarte, Liliane Ventura

**Affiliations:** Department of Electrical and Computing Engineering, São Carlos School of Engineering, University of São Paulo, Av. Trabalhador Sancarlense 400, São Carlos, SP 13566-590 Brazil

**Keywords:** Biophysics, Engineering

## Abstract

The media and even the specialized literature report that the ultraviolet (UV) protection for sunglasses is critical, on the grounds that sunglasses can have a counter effect if the lenses do not provide adequate UV protection. They reason that the primary and natural mechanism is that the pupil of the eye contracts to attenuate radiation and protect the inner eye under sun exposure. Therefore, if dark lenses do not provide appropriate UV protection, there is an increased UV incidence in the inner eye due to pupil dilation, which enhances the adverse effects and impacts the ocular tissues more severely than in situations without UV protection. However, no existing literature properly quantified or supported this argument. In this work, the influx of solar UV throughout the pupil of the eye was calculated in two situations: when a person wear sunglasses and when he/she does not. In both situations, the pupil dilation and the field of view (squint) were considered with their dependence on the brightness of the ambient, calculated by modeling the solar irradiation. Finally, it was assessed whether sunglasses with poor UV protection actually increase the UV influx throughout the dilated pupil compared to the non-dilated pupil. A set of 214 sunglasses lenses were tested and the results show that pupil dilation does not play an important role in the UV influx throughout the pupil. It was observed that the FOV is the main player, surpassing the pupil size contribution by up to 314.3%, disproving the common explanation. Because of the major role of the FOV, our results show that sunglasses with UV-A protection below 86% may have a slight potential to increase hazards to the eye compared to not wearing sunglasses at all. These results can have direct impact on sunglasses standards regarding the UV protection linked to the category of the lenses.

## Introduction

The primary purpose of sunglasses is to dim visible light to comfortable levels of luminance, glare, and retinal light-saturation. In addition, sunglasses are designed to filter out harmful ultraviolet (UV) radiation. An assortment of branded and unbranded sunglasses can be found worldwide and a number of them do not efficiently filter out part of the UV range.

One of the hotly debated topics regarding sunglasses and eye protection against solar UV radiation is whether low quality, informal (unbranded) economy sunglasses in the market are harmful to eye health if they do not provide a proper filter nor are certified to meet current standards. When an individual is exposed to sunlight, the pupil reflexively constricts. When wearing sunglass lenses, due to the reduced amount of light transmitted through the lenses, the pupil will not constrict to the same extent. If the sunglass lenses have insufficient UV radiation filtering capability, then hypothetically, the eyes will be exposed to higher levels of UV radiation. Entities that advocate for strict product standards frequently make this argument, including a very recent publication^1–4^.

The marketing for the consumer to purchase certified sunglasses that are UV protected has done a very good job in alerting the public, and it publicizes the information that it is better to go outdoors in the sun without sunglasses rather than with sunglasses that do not provide proper UV protection^1^. However, the subject remains contradictory in some aspects. The question that persists is whether the materials of sunglasses lenses, because of their intrinsic properties, absorb enough UV radiation to prevent its influx into the eye due to the pupil dilation triggered by the sunglasses. Hoover addressed this problem^5^ and made a great effort to perform theoretical calculations of the geometry of the eye when a person is outdoors with and without sunglasses, taking into account the spectral transmittances of sunglasses. Nevertheless, Hoover considered only a few situations, and the lack of an extensive calculation remains. In addition, Hoover took into account in his calculations the UV upper limit as 380 nm. However, UV radiation is often defined with an upper limit of 400 nm, which is more appropriate^6–11^. Hoover showed that for specific situations of luminance and using the action spectrum of UV radiation and the eye, the argument could break down, i.e., even low-quality sunglasses would protect against UV radiation, regardless of how much pupil dilation stemmed from wearing such sunglasses. Although in his previous work^12^, he pointed out that squint would be a dominant response of a person in a bright environment, in his subsequent work^5^, this effect was not taken into account. He considered a fixed cone with a 55° half-angle as the field of view (FOV). Sliney et al.^13,14^ showed that the FOV is fairly related to ambient luminance, with a quantitative analysis^13,14^ only available much after Hoover’s work^5^. Another concern related to sunglass lenses is the degradation of their UV protection after long-term irradiation within solar simulators ^15^, posing an additional risk to the wearer. This concern was raised following an investigation demonstrating that the resistance to the irradiation test, required by the standards, is ineffective and does not provide assurance for long-term UV protection^16^.

Beyond the protective capacity provided by sunglass lenses, the literature indicates that it is not only direct sunlight exposure to the eyes that should be a concern, but also the radiation incident from the sides^17–23^. Ophthalmic epidemiological studies^24–26^ have established a connection between cortical cataracts and the limbal focusing of peripheral UV rays onto the germinative area of the crystalline lens, known as the Coroneo effect^18–20,22^. Coroneo’s observations indicated that peripheral rays can focus in the nasal lens sector. Consequently, UV rays from sunglasses without side shields can pose risks to the crystalline lenses. Therefore, for optimal protection against UV radiation, it is essential to consider the entire frame design, not just the lens. The inclusion of side shields and overhead protection is advised^17,21,23^.

This work aims to undertake an extensive, meticulous, and detailed analysis, with theoretical evaluations and experimental data, to advance the elucidation of this problem. This study intends to show which is best for ocular safety, in addition to using good quality sunglasses: wearing poor-quality sunglasses or avoiding them. It should take into account a better model of solar spectral irradiance and sky luminance. Additionally, it should use an improved calculation of the pupil diameter, and most importantly, to consider the FOV depending on the environmental luminance. It should be stressed that this investigation does not concern UV radiation coming from the back nor sideways, that is, the incidence of radiation on the inner surface of the sunglass lenses is neglected. In this situation, the frame design plays the most important role in temporal peripheral rays^17,21,23^.

In what follows, we provide a detailed description of the method, both theoretical and experimental parts, and discuss the most significant results that support our conclusions.

### Theoretical models and experimental method

**Figure 1:**
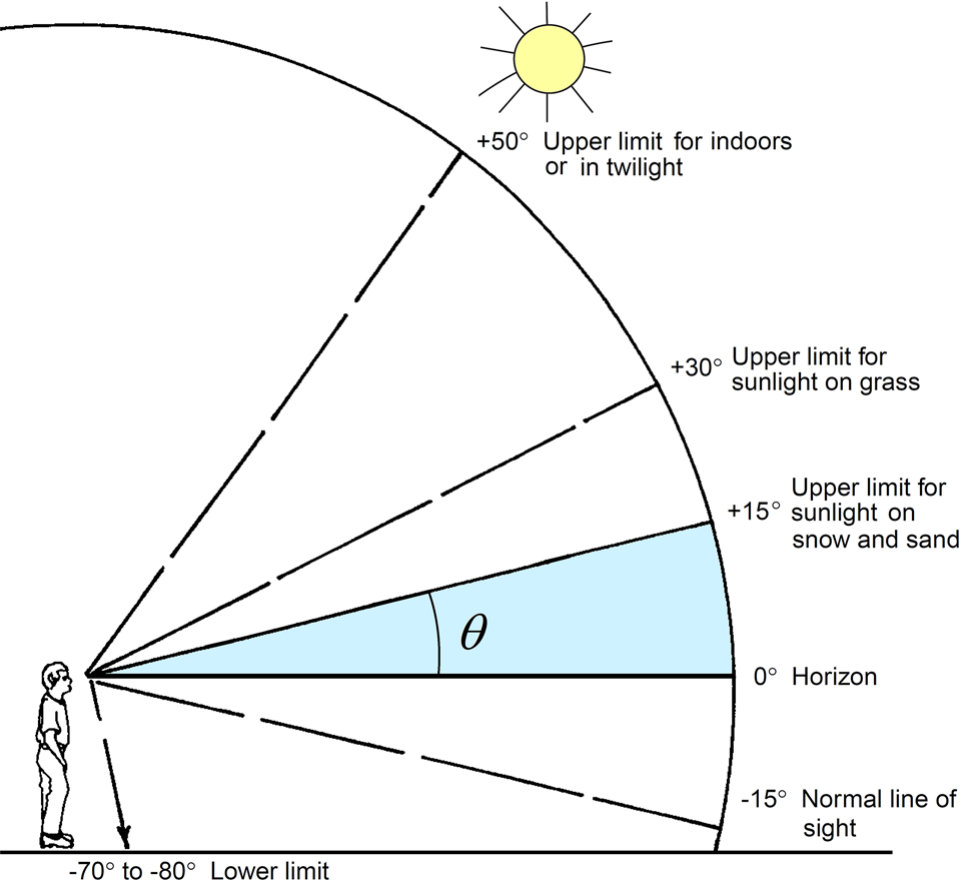
Geometric aspects related to the position of the Sun and the individual with a line of sight to the horizon. (adapted from Sliney^27^).

The typical situation that this paper proposes to investigate is that of a person wearing sunglasses in an upright position exposed to atmospheric conditions described as clear sky, i.e., without clouds and pollutants. This person can be anywhere in the world, on any day or time of the year. In this scenario, the person will keep a flat ($0^\circ$) line of sight toward the sun’s horizontal projection, as outlined in Fig. 1 (adapted from Sliney^27^).

In the depicted scenario, we calculate solar irradiance and radiant exposure on a vertical surface by simulating the solar radiation spectrum with a widely used model validated in the literature^28–31^. As a practical example, this calculation will be carried out for the latitude of the city of São Paulo (− 23°32′51″ S), for every day of the year, from sunrise to sunset. It is worth mentioning that Hoover’s calculation^5^ considered the half-angle cone as $\theta = 55^\circ$, which is above the upper limit for direct irradiance even indoors, according to Fig. 1.

### Solar irradiance

Global solar irradiance (W/m^2^) is composed of direct, diffuse, and reflected irradiances. Taking the UV range, the total direct irradiance on a vertical surface, denoted by $E_{b}$, for a given time and at a given location, is calculated in terms of the solar spectral irradiance by:

1 $$E_{b} \left( {{\mathbf{r}},t} \right) = \mathop \smallint \limits_{{280 {\text{nm}}}}^{{400 {\text{nm}}}} E_{b} \left( {\lambda ,{\mathbf{r}},t} \right){\text{sin}}\left[ {Z_{S} \left( {{\mathbf{r}},t} \right)} \right]d\lambda ,$$

in which ${\mathbf{r}}$ represents geographic coordinates (latitude, longitude, and altitude) and $t$ represents time coordinates (days of the year and time of day). The angle $Z_{S} \left( {{\text{r}},t} \right)$ is the solar zenith angle as a function of location and time. The total diffuse irradiance, denoted by $E_{d}$, is calculated by a similar equation. In Eq. (1), the solar spectral irradiance $E_{b} \left( {\lambda ,{\mathbf{r}},t} \right)$ at ground level should be obtained either by measurements or by theoretical modeling. Once collected data on solar spectral irradiance are not readily available, one must use an atmospheric model. Attempts to model the solar spectrum at ground level can be found in the literature^12,32^. In this paper, we use a model called the *Simple Model of the Atmospheric Radiative Transfer of Sunshine*, *Version 2* (SMARTS2)^28–30^. This model is based on satellite data and atmospheric radiative transfer theory. The choice of this model is based on its reliability, accuracy, and availability of open-source codes that can be adapted to local conditions. Additionally, this model is accurate when compared with proprietary models and other methods in the literature^30,31^.

### Sky luminance

**Figure 2:**
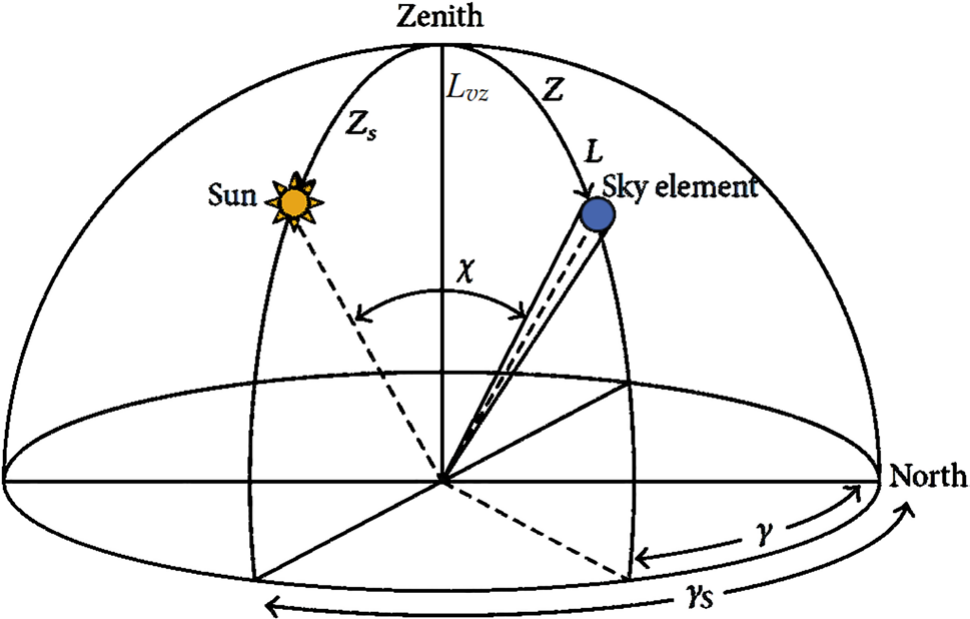
Geometry related to the position and a given sky element in the luminance calculation.

Given the condition outlined in Fig. 1, the luminance $L$ of a sky element will be calculated as described in Fig. 2, in which the main geometric variables for the luminance calculation are represented. Namely, $Z$ is the zenith angle, $\gamma$ is the azimuth angle (from north and clockwise), and $\chi$ is the angular distance between the Sun and the element of the sky. The subscript “$S$” in the variables stands for the Sun.

The angular distance $\chi$ between the sun and the sky element is given by:

2 $$\cos \chi = \cos Z_{S} \cos Z + \sin Z_{S} \sin Z\cos \left( {\left| {\gamma - \gamma_{S} } \right|} \right).{ }$$

In the calculation of the luminance of the sky element, we shall use a luminance distribution model^33^, which has been proven an accurate model among the best available models in the literature. In this model, the luminance is calculated in terms of the illuminance, which in turn is attained from the solar spectral irradiance. However, unlike Ref.^33^, in this work, we calculate the solar irradiance by using the SMARTS2 model^28–30^, which is more accurate. The luminance $L_{va}$ of a sky’s element is calculated by:

3 $$L_{va} \left( {Z_{S} ,Z,\chi } \right) = L_{vZ} \left( {Z_{S} } \right)L_{v} \left( {Z_{S} ,Z,\chi } \right).$$

The function $L_{v} \left( {Z_{S} ,{\text{Z}},\chi } \right)$ is the relative luminance distribution of the sky, calculated by the product of the gradation function $\phi \left( Z \right)$ by the indicatrix function $f\left( \chi \right)$, i.e.,

4 $$L_{v} \left( {Z_{S} ,Z,\chi } \right) = \frac{\phi \left( Z \right)f\left( \chi \right)}{{\phi \left( 0 \right)f\left( {Z_{S} } \right)}},$$

in which $\phi \left( Z \right)$ and $f\left( \chi \right)$ are given by:

5 $$\phi \left( Z \right) = 1 + A e^{ - B/\cos Z}$$

and

6 $$f\left( \chi \right) = 1 + C\left[ { e^{ - D\chi } - e^{ - D\pi /2} } \right] + E\cos^{2} \chi .$$

The constants for a clear sky condition are given in Ref.^34^ and are $A = - 1$, $B = 0.55$, $C = 10$, $D = 3$, and $E = 0.45$. The phase function represented by Eq. (6) is the angular intensity of the forward scattering.

In Eq. (3), $L_{vZ} \left( {Z_{S} } \right)$ is the zenith luminance, given by:

7 $$L_{vZ} \left( {Z_{S} } \right) = \frac{{E_{vd} }}{{\mathop \smallint \nolimits_{Z = 0}^{\pi /2} \mathop \smallint \nolimits_{\gamma = 0}^{2\pi } L_{v} \left( {Z_{S} ,Z,\chi } \right)\sin Z\cos Z\;dZ\;d\gamma }},$$

in which ${E}_{vd}$ is the diffuse illuminance and will be computed using the SMARTS2 model^28–30^ instead of the method given in Ref.^33^, as mentioned earlier.

In the case depicted in this work, the azimuth angle $\gamma$ of the sky element is equal to the sun’s azimuth angle $\gamma_{S}$. The angular distance $\chi$ between the sun and the sky element is equal to the sun elevation, which is $90^\circ - Z_{S}$ (see Fig. 2). This means that the elevation of the sky element is $0^\circ$ ($Z = 90^\circ$), that is, line of sight on the horizon.

As pointed out by Gueymard and Ivanova^35^, the Igawa model^33^, used in this work, underestimates the circumsolar radiance due to inaccuracies in radiance measurements around the Sun. Hence, to avoid these inaccuracies, the angular distance between the sun center and the sky element should be greater than $18^\circ$ for reliable modeling of the luminance. Therefore, this angular distance constraint will be respected in the present investigation.

### Pupil diameter

**Figure 3:**
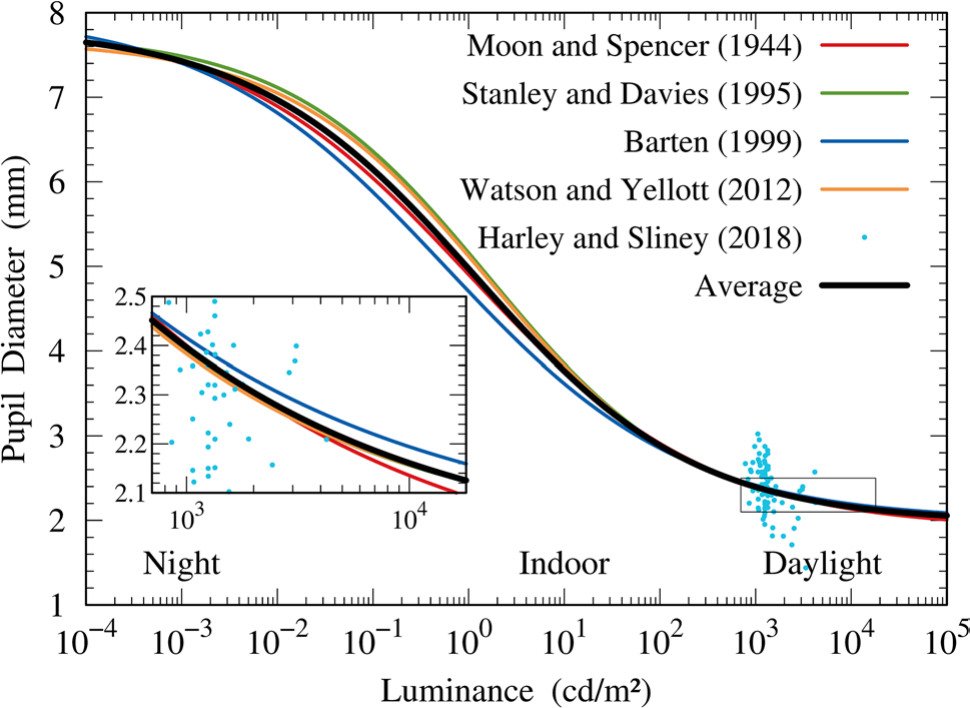
A comparison is presented for four models^38–41^ concerning pupil diameter, along with their respective average. Additionally, the experimental data are shown^42^. The inset illustrates the range of interest in the current calculation of luminance and pupil diameter.

Pupilometry studies have been conducted since 1918^36,37^ to estimate the average pupil size over a range of luminance levels ranging from scotopic to photopic levels. These experiments measured the pupil size depending on ambient luminance and subsequently, a mathematical function was adjusted to fit the data measurements. Watson and Yellott^38^ provided a comprehensive review of commonly used models in pupilometry. In addition, they developed their model for the diameter of the pupil. Among the models reviewed in Ref.^38^, we selected three models^39–41^ that give results in best agreement with the model of Ref.^38^. These models have different experimental measurements on different subjects. Therefore, to avoid favoring any of the four models^38–41^ in particular and to pursue a combined result among them, we used a postprocessing ensemble model to estimate an optimal expectation for the diameter of the pupil, given the estimates of the four selected models. This ensemble model estimate should cancel out the individual models’ biases at some level. Because the pupil area is not critical in the present investigation, naïve ensemble modeling simply calculates the respective arithmetic mean. The mathematical functions of each selected model are summarized in Ref.^38^, and their average is shown in Fig. 3. Therefore, when needed, the results of the ensemble model for the diameter of the pupil will be used. Harley and Sliney^42^ conducted a more recent experimental assessment of pupil size in outdoor environments. The results of their experiments are depicted in Fig. 3, showing that the observed pupil sizes exhibit great variability amongst individuals. However, the results align with the chosen models. Hence, the current model is supported by more recent experimental findings.

### Field of view (FOV)

In very bright environments, such as outdoors in daylight, there are natural and involuntary facial features and reactions aiming to attenuate the illuminating radiation in the eye media. For instance, brow ridges, eyebrows, eyelids, and squinting bring down the solid angle of light admittance into the eye. The solid angle of the light admittance corresponds to half of the apex angle of a right cone, i.e., the apex angle is $2\theta$. The angle $\theta$ (see Fig. 1) is the FOV and is related to the corresponding solid angle ${\Omega }$ (in sr) by

8 $${\Omega } = 4\pi \sin^{2} \left( {\frac{\theta }{2}} \right).$$

Sliney and co^13,14^ investigated how the ambient luminance determines the FOV and reached an empirical expression that fairly models the FOV, given by

9 $$\theta = 34^\circ - 0.0013L_{va} ,$$

in which $L_{va}$ is the ambient luminance given in cd/m^2^ and calculated by Eq. (3). This expression shall be used here to estimate the light admittance cone in the pupil, with and without sunglasses.

### UV influx

In the situation under consideration (Fig. 1), one calculates the luminance of a sky element (Fig. 2) and the associated pupillary diameter and FOV. Hence, one can calculate the solar radiation admittance throughout the eye pupil that reaches the crystalline lens. In this sense, two calculation conditions are presented. One condition refers to the situation in which a person wears sunglasses and the other one without sunglasses. Thus, the UV influx through the eye pupil can be calculated under both conditions. The ratio between these two results gives the relative influx, denoted $\rho_{UV}$, i.e.,

10 $$\rho_{UV} = \frac{{{\Omega }_{u} }}{{{\Omega }_{c} }}\frac{{A_{u} }}{{A_{c} }}\frac{{\mathop \smallint \nolimits_{{280 {\text{nm}}}}^{{400{\text{nm}}}} E_{i} \left( \lambda \right)\tau_{e} \left( \lambda \right)\tau_{sg} \left( \lambda \right)d\lambda }}{{\mathop \smallint \nolimits_{{280 {\text{nm}}}}^{{400{\text{nm}}}} E_{i} \left( \lambda \right)\tau_{e} \left( \lambda \right)d\lambda }} = \omega \alpha \frac{{\mathop \smallint \nolimits_{{280 {\text{nm}}}}^{{400{\text{nm}}}} E_{i} \left( \lambda \right)\tau_{e} \left( \lambda \right)\tau_{sg} \left( \lambda \right)d\lambda }}{{\mathop \smallint \nolimits_{{280 {\text{nm}}}}^{{400{\text{nm}}}} E_{i} \left( \lambda \right)\tau_{e} \left( \lambda \right)d\lambda }}.$$

**Figure 4:**
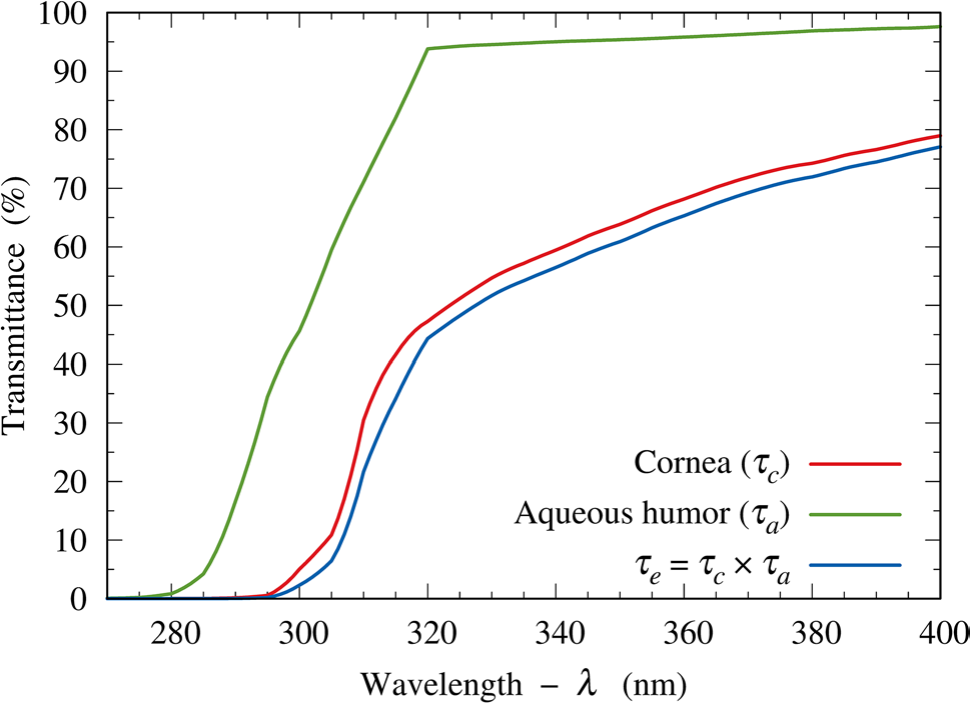
Spectral transmittances of the anterior components of the eye^43^ and the product $\tau_{e} \left( \lambda \right)$.

In Eq. (10), the incident solar spectral irradiance on the cornea, $E_{i} \left( \lambda \right)$, is calculated using the SMARTS2 model. $\tau_{e} \left( \lambda \right)$ is the combined spectral transmittance of the eye components anterior to the surface of interest. For a flat pupil surface, $\tau_{e} \left( \lambda \right)$ comprises the product of the corneal and aqueous humor spectral transmittances. Both transmittances are described in the literature^43^ and shown in Fig. 4. The spectral transmittance of sunglass lenses, denoted by $\tau_{sg} \left( \lambda \right)$, will be considered in the next subsection. The values $A_{u}$ and $A_{c}$ are the pupil areas with and without sunlight attenuating lenses, respectively, i.e., $A_{u}$ is the unconstricted pupil area and $A_{c}$ is the constricted pupil area. The angles ${\Omega }_{u}$ and ${\Omega }_{c}$ denote the solid angles corresponding to the FOV for each condition. The product $\omega \alpha$ is the overall combined contributions of the pupil areas and the solid angles.

From Eq. (10), a relative influx greater than the unit $\left( {\rho_{UV} > 1} \right)$ means that the darker environment produced by the sunglasses triggered greater FOV and pupil dilation, allowing a greater incidence of UV radiation to the inner eye than the UV influx without sunglasses. In contrast, $\left( {\rho_{UV} < 1} \right)$ implies that the FOV and pupil dilation due to wearing sunglasses are not sufficient to promote an increase in UV influx throughout the pupil compared to influx without sunglasses.

### Sunglasses lenses spectroscopy

The spectral transmittances of the sunglass lenses, $\tau_{sg} \left( \lambda \right)$, should be measured to be included in Eq. (10). Although this investigation focuses on UV radiation, transmittances were measured in the UV–Vis range, i.e., 200–800 nm. We used a double beam UV–Vis CARY 5000 spectrophotometer (AGILENT) and a UV–Vis 1800 (SHIMADZU). All lenses were measured spectroscopically on both spectrophotometers using a circular mask with a 5 mm diameter in the center of the lenses. The spectral resolution was 1 nm. Before each measurement, the lenses were cleaned to avoid spurious interference.

## Results and discussion

For our purposes, we have chosen as a typical example for all calculations the Brazilian city of São Paulo, which is located at latitude − 23°32′51″ S, longitude − 46°38′10″ W, and a mean altitude of 760 m. A clear sky assumption has also been made because it is the worst-case scenario for exposure to the sun. All integrals in the equations were computed using the 5-point Gauss–Legendre quadrature method and Lagrange’s cubic interpolation of the integrand.

Our calculations were carried out for each day of the year, from sunrise to sunset, but respecting the minimum of $18^\circ$ for sun elevation, as pointed out earlier. In the calculation of the UV influx in the eye, we reported here only the results for the highest exposure day of the year, which is day number 356 (December 21).

The present investigation used a set of 214 lenses (three made of glass and the remaining made of plastic materials) of unbranded sunglasses, ranging from category 1 (lighter tint) to category 4 (darker tint) and without category (darker than allowed by the ISO standard, i.e., luminous transmittance below 3%)^44^. Hereafter, we shall label the latter as “non-category”. In this sample, 6 were category 1, 109 were category 2, 65 were category 3, 26 were category 4, and 8 were “non-category”. Lenses from category 0 were not investigated because they are clear lenses and do not induce pupil dilation. Unbranded sunglasses were donated by ABIÓPTICA (the Brazilian association of sunglasses industries). They did not have any prior information about their specifications.

Out of the 214 tested lenses, 8 presented a higher risk when worn than otherwise unprotected eyes. Table 1 shows the worst-case results for each lens category, comprising the 8 unsafe lenses, which appear in boldface. At first, all lenses in category 1 are suitable for wear because they are too light and do not provide enough light attenuation to induce significant pupil dilation and greater FOV. All lenses in category 2 with UV-A protection greater than 81% and in category 3 over 86% exhibited effective protection. The hazardous lenses for the eye are of categories 2 and 3. Note that UV-B protection appears to have negligible significance, as discernible patterns do not emerge from the corresponding column in Table 1. Although it could be expected that the most dangerous lenses would be in categories 4 and non-category (too dark), all 34 lenses (26 lenses of category 4 and 8 lenses of non-category) in these two categories had at least 88% UV-A protection and therefore are suitable to wear. As an example of the best lenses, Table 1 lists one lens with 100% UV protection, marked in italics (label R311-24). The last column of Table 1 shows the pass/fail test as required by the ISO standard^44^. Notice that only one lens is compliant with the standard. However, it is interesting to note that even failed lenses (by the current standard) can still be protective, although not in the best sense. This means that wearing those failed lenses is better than not wearing them at all.

**Table 1:** Selected lenses in categories 1–4 and non-category, showing the safe and hazardous lenses (marked as boldface).

Lens label	Category	${\tau }_{V}$ (%)	UV protection (%)	UV-A protection (%)	UV-B protection (%)	Compliance
R210-08	1	43.433	83.810	77.931	93.341	Fail UV-B
R273-06	1	43.140	84.398	78.631	93.750	Fail UV-B
**R139-09**	**2**	**18.683**	**76.328**	**68.327**	**89.299**	**Fail UV-A/UV-B**
**R268-06**	**2**	**40.586**	**80.637**	**74.324**	**90.863**	**Fail UV-A/UV-B**
**R197-08**	**2**	**38.927**	**83.425**	**75.886**	**95.645**	**Fail UV-A/UV-B**
**R080-08**	**2**	**29.035**	**85.675**	**77.216**	**99.389**	**Fail UV-A**
**R126-22**	**2**	**23.907**	**87.977**	**80.560**	**99.999**	**Fail UV-A**
**R071-01**	**2**	**27.075**	**86.255**	**80.936**	**94.879**	**Fail UV-A/UV-B**
R275-05	2	42.241	86.901	81.647	95.416	Fail UV-B
R362-06	2	25.577	88.037	85.552	92.065	Fail UV-A/UV-B
**R069-11**	**3**	**12.453**	**78.609**	**70.767**	**91.320**	**Fail UV-A/UV-B**
**R075-20**	**3**	**9.632**	**90.905**	**85.341**	**99.922**	**Fail UV-A**
R195-20	3	17.937	91.402	87.251	98.131	Fail UV-A/UV-B
R010-06	3	17.512	90.564	88.412	94.056	Fail UV-A/UV-B
R124-01	4	7.885	92.070	88.000	98.669	Fail UV-A/UV-B
R144-11	4	7.532	96.782	94.863	99.894	Fail UV-A
*R311-24*	*4*	*3.291*	*100.000*	*100.000*	*99.999*	*Passed*
L305-18	–	2.492	99.989	99.987	99.993	–
R224-18	–	2.641	99.991	99.988	99.997	–

One ideal lens with 100% UV protection (marked in italics) is listed. $\tau_{V}$ is the luminous transmittance (400–780 nm).

The UV-A and UV-B protection requirements are defined by the lens category and the luminous transmittance ($\tau_{V}$) itself, as listed in Table 2, which is adapted from the ISO 12312-1 standard^44^.

**Table 2:** UV protection requirements for sunglass lenses for general use, adapted from the ISO 12312-1 standard^44^.

Category	Requirements		
	Ultraviolet spectral range		Visible spectral range
	Maximum value of solar UV-B transmittance (280–315 nm)	Maximum value of solar UV-A transmittance (315–400 nm)	Range of luminous transmittance $\tau_{V}$ (400–780 nm)
0	$0.05\tau_{V}$	$\tau_{V}$	$\tau_{V} > 80\%$
1	$0.05\tau_{V}$	$\tau_{V}$	$43\% < \tau_{V} \le 80\%$
2	$\max \left( {1\% ,0.05\tau_{V} } \right)$	$0.5\tau_{V}$	$18\% < \tau_{V} \le 43\%$
3	$1\%$	$0.5\tau_{V}$	$8\% < \tau_{V} \le 18\%$
4	$1\%$	$\max \left( {1\% ,0.25\tau_{V} } \right)$	$3\% < \tau_{V} \le 8\%$

$\tau_{V}$ is the luminous transmittance (400–780 nm).

**Figure 5:**
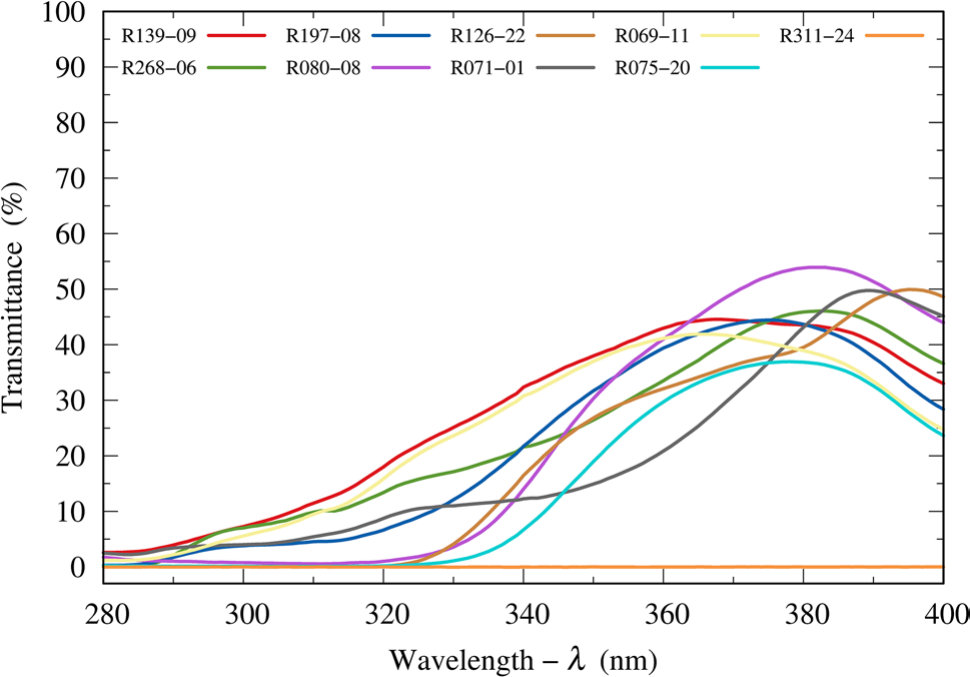
Ultraviolet spectral transmittances for the 8 worst sunglass lenses and 1 lens filtering up to 400 nm (R311-24).

Figure 5 shows the measured UV spectral transmittances of the 8 worst lenses and 1 ideal lens (100% UV protection) listed in Table 1. They have significant UV transmission, except for the ideal transmission, which has a flat spectral transmittance of 0%.

**Figure 6:**
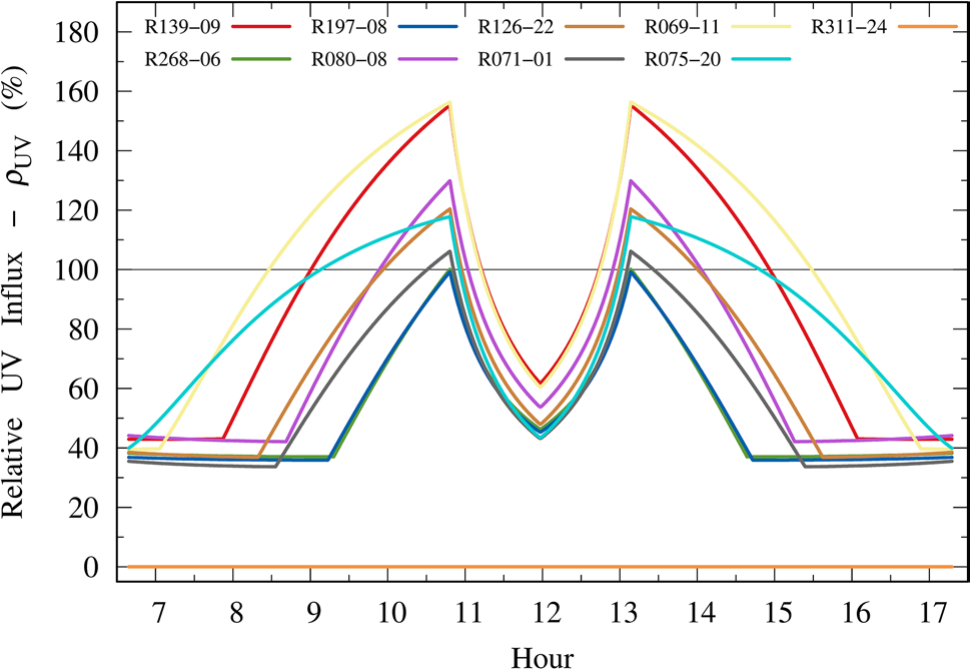
Relative UV influx of 8 failed lenses and 1 ideal lens.

Figure 6 shows the UV relative influx $\rho_{UV}$ (Eq. 10) for the lenses shown in Fig. 5. All curves are for day number 356 (December 21). The harmful periods are those with $\rho_{UV}$ greater than 100%, which means that sunglasses would allow more UV influx than influx with no sunglasses.

**Figure 7:**
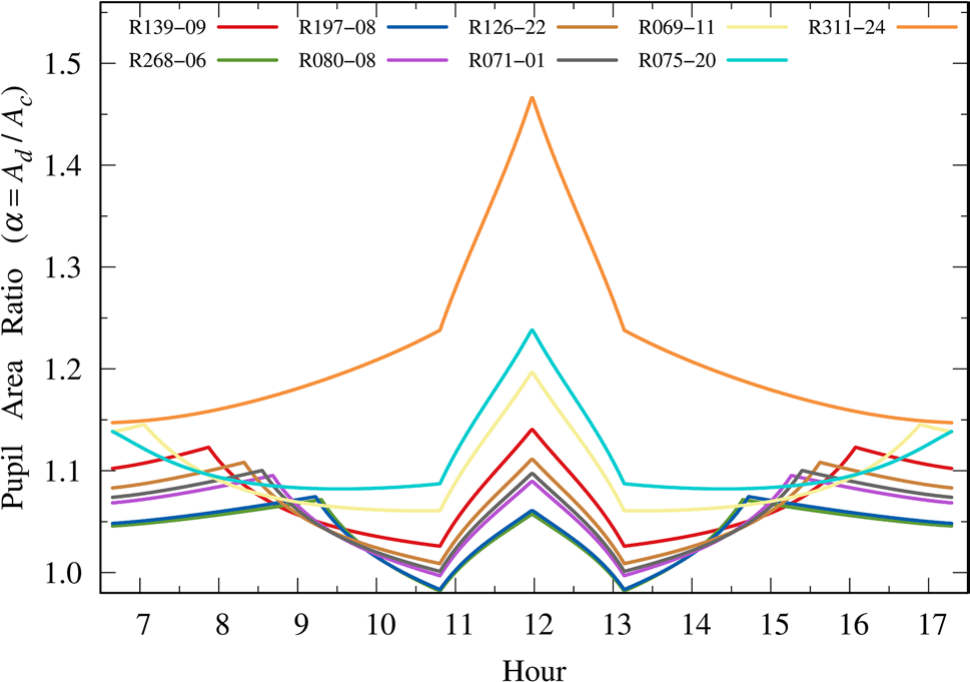
Pupil area ratio between unconstricted (with sunglasses) and constricted (without sunglasses) pupils.

The main factor in this behavior is the FOV ratio ($\omega$) with and without sunglasses. The pupil area ratio ($\alpha$) plays a minor role and by itself is not accountable for the present results (Eq. 10). For instance, Fig. 7 shows the pupil area ratio for the selected lenses. Note that the darkest lens (R311-24) has the greatest ratio at all times, peaking at approximately noon at a value of 1.47. However, this lens has 100% UV protection. For the worst lens (R069-11), the peak in the pupil area ratio $\alpha$ around noon is only 1.20, which is not enough to result in a hazard to the eye.

**Figure 8:**
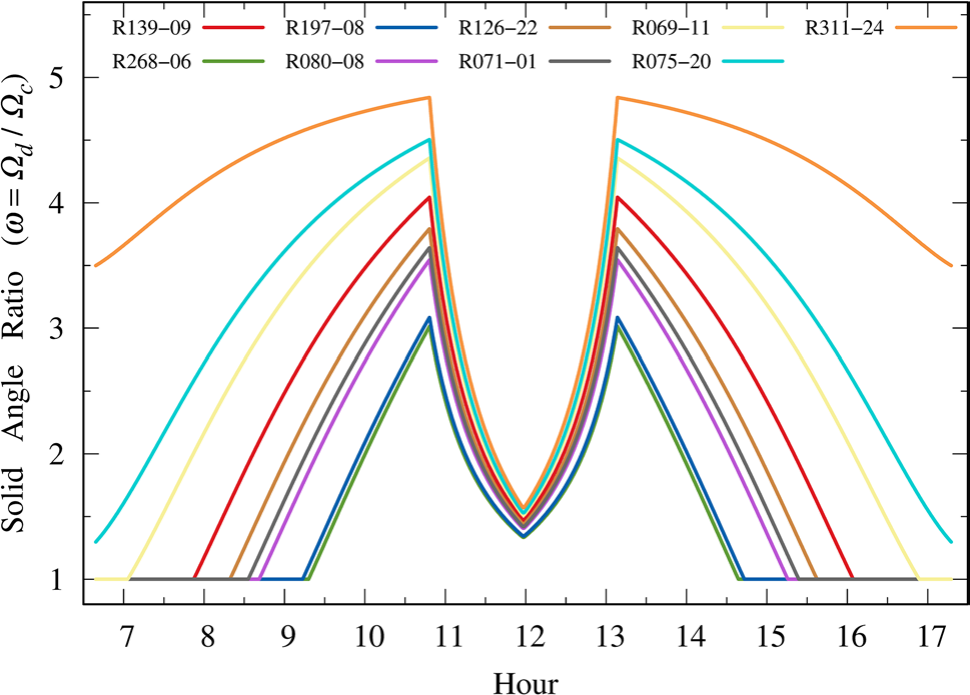
Solid angle ratio comprising the FOV with and without sunglasses. The darker the lens, the greater the ratio.

Conversely, the FOV ratio plays a major role, as shown in Fig. 8. Compared with Fig. 7, the ratio $\omega$ is much greater than the ratio $\alpha$. This behavior shows a dip in $\rho_{UV}$ around noon, as depicted in Fig. 6. The decline in the FOV ratio $\omega$ (refer to Eq. 10) stems from the expanding FOV affecting the denominator of $\omega$, i.e., the FOV without the use of sunglasses. Subsequently, under high sun elevation conditions when there is no direct irradiance on the eyes, the attenuation in the squint response results in the denominator of $\omega$ increasing at a faster rate than the numerator. This dynamic causes the $\omega$ ratio to decrease, as illustrated in Fig. 8.

**Figure 9:**
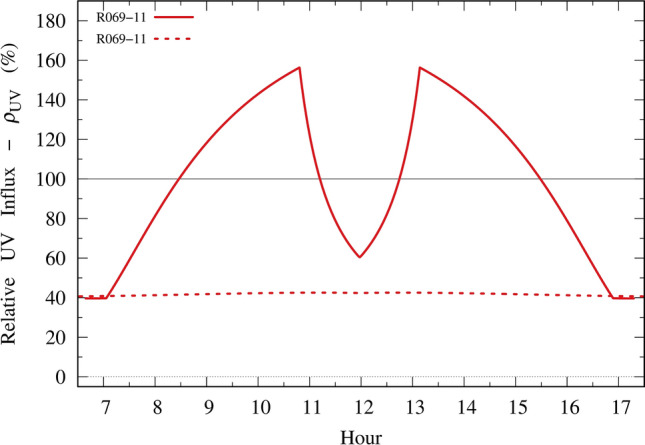
Comparison between the relative UV influx to the eye taking into account the FOV ratio (squint response) and the pupil dilation ratio, with the result neglecting the FOV ratio. Solid line: with $\alpha$ and $\omega$. Dotted line: without $\omega$.

This effect is evident in the relative UV influx $\rho_{UV}$. For example, considering the worst-case (R069-11), if one completely disregards the FOV ratio $\omega$ and takes into account only the ratio $\alpha$, the results present a noticeable difference. This outcome is shown in Fig. 9, in which the solid line represents the full calculation of the relative influx $\rho_{UV}$ (considering $\alpha$ and $\omega$) and the dotted line represents the calculation neglecting the FOV ratio $\omega$. This latter result would infer a false acceptable lens, while it is a very poor and hazardous lens, allowing more UV influx to the eye if not used at all.

**Figure 10:**
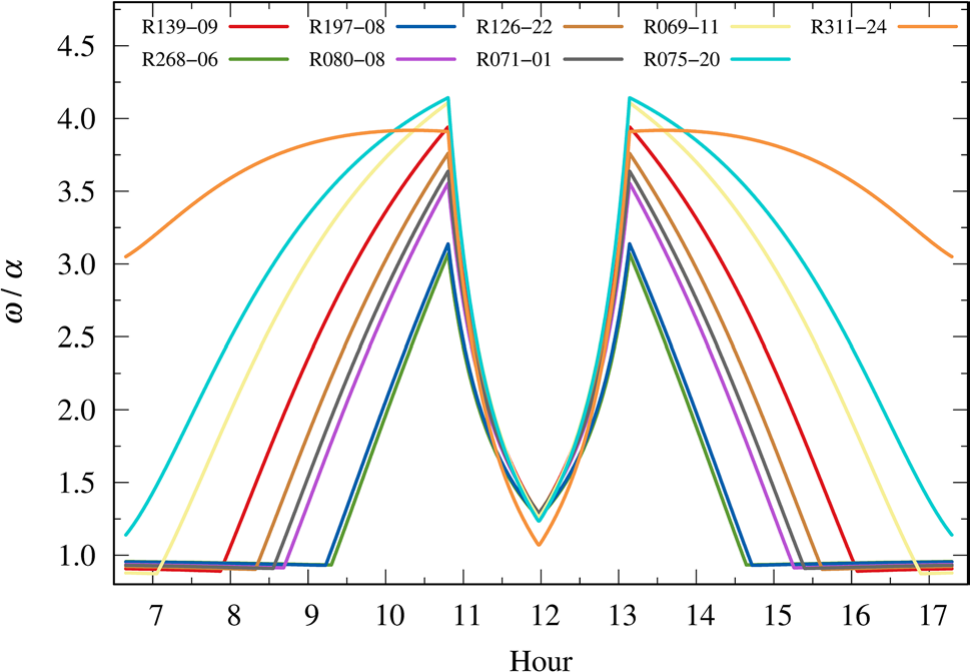
Ratio between the FOV contribution ($\omega$) to the pupil size contribution ($\alpha$) to the UV influx in the inner eye.

The ratio between the FOVs ($\omega$) is generally greater than the ratio between the pupil sizes ($\alpha$) most of the time, as depicted in Fig. 10. In this figure, during the early morning and late afternoon, some lenses exhibit $\alpha > \omega$, indicating that pupil dilation has a greater contribution to the influx of UV radiation than the FOV, given that $\omega /\alpha$ is less than unity. However, the contribution of pupil dilation during these periods is marginal, considering that $\omega /\alpha$ is slightly less than unity. On the other hand, during periods when the FOV has a more relevant contribution, $\omega$ can be as high as 4.143 times (314.3%) greater than $\alpha$.

## Conclusion

In summary, all lenses with UV-A protection below 86% exhibited a potential risk, as they allow more UV influx in the eye than they would have if the sunglasses were not used. Our results support the main conclusion that wearing sunglasses with poor UV protection may worsen the hazards to interior eye health. However, the results establish that the reason for this worsening is not pupil dilation triggered by sunglasses. The more appropriate argument is that the field of view is small in a bright outdoor environment due to the involuntary squint response, posing a natural attenuation of the solar radiation influx into the eye. Sunglasses provide a darker environment, hampering natural involuntary lid responses and therefore increasing the field of view. In this context, the objectives of this investigation have been achieved, i.e., sunglasses with poor UV protection allow more UV radiation influx in the eye than influx due to the smaller field of view without sunglasses. Since the present results were obtained for UV up to 400 nm, we suggest that manufacturers should strive to not simply meet minimum standards but to achieve UV400 properties for all sunglass lenses to mitigate any risk of unnecessary UV radiation exposure.

## Data Availability

The main calculations used the SMARTS2 codes, available from ref.^28^.

## Abbreviations

CIE: Commission Internationale de l’Eclairage (International Commission on Illumination)
FOV: Field of View
ICNIRP: International Commission on Non-Ionizing Radiation Protection
ISO: International Organization for Standardization
SMARTS2: Simple Model of the Atmospheric Radiative Transfer of Sunshine version 2
UV: Ultraviolet
